# Traditional Mexican dietary pattern and cancer risk among women of Mexican descent

**DOI:** 10.1007/s10552-024-01849-5

**Published:** 2024-02-02

**Authors:** Nicole C. Loroña, Margarita Santiago-Torres, Melissa Lopez-Pentecost, Lorena Garcia, Aladdin H. Shadyab, Yangbo Sun, Candyce H. Kroenke, Linda G. Snetselaar, Marcia L. Stefanick, Marian L. Neuhouser

**Affiliations:** 1 Fred Hutchinson Cancer Center, Seattle, WA USA; 2 Department of Epidemiology, University of Washington, Seattle, WA USA; 3 Sylvester Comprehensive Cancer Center, University of Miami Miller School of Medicine, Miami, FL USA; 4 Department of Public Health Sciences, Division of Epidemiology, UC Davis School of Medicine, Davis, CA USA; 5 Herbert Wertheim School of Public Health and Human Longevity Science, University of California, San Diego, La Jolla, CA USA; 6 The University of Tennessee Health Science Center, Memphis, TN USA; 7 Division of Research, Kaiser Permanente Northern California, Oakland, CA USA; 8 Department of Epidemiology, College of Public Health, University of Iowa, Iowa City, IA USA; 9 Department of Medicine (Stanford Prevention Research Center), Stanford University School of Medicine, Stanford, CA USA

**Keywords:** Mexican diet, WHI, Breast cancer, Colorectal cancer, Dietary pattern, Cancer risk

## Abstract

**Purpose:**

To examine the association of a traditional Mexican diet score with risk of total, breast, and colorectal cancer among women of Mexican ethnic descent in the Women’s Health Initiative (WHI).

**Methods:**

Participants were WHI enrollees who self-identified as being of Mexican descent. Data from food frequency questionnaires self-administered at study baseline were used to calculate the MexD score, with higher scores indicating greater adherence to an a priori-defined traditional Mexican diet (high in dietary fiber, vegetables, and legumes). Incident cancers were self-reported by participants from 1993 to 2020 and adjudicated by trained physicians. We used multivariable-adjusted Cox proportional hazards models to calculate hazard ratios (HRs) and 95% confidence intervals (CIs).

**Results:**

Among 2,343 Mexican descent women (median baseline age: 59 years), a total of 270 cancers (88 breast, 37 colorectal) occurred during a mean follow-up of 14.4 years. The highest tertile of MexD score was associated with a lower risk of all-cancer incidence (HR: 0.67; 95% CI 0.49–0.91; p-trend: 0.01) and colorectal cancer (HR: 0.38; 95% CI 0.14–0.998; p-trend < 0.05), with each unit increase in the MexD score associated with a 6% lower risk of all-cancer incidence (HR: 0.94; 95% CI 0.88–0.99). There was no statistically significant association with risk of breast cancer.

**Conclusion:**

Consumption of a traditional Mexican diet was associated with a significantly lower risk of all-cancer incidence and colorectal cancer. Confirmation of these findings in future studies is important, given the prevalence of colorectal cancer and a growing U.S. population of women of Mexican descent.

**Supplementary Information:**

The online version contains supplementary material available at 10.1007/s10552-024-01849-5.

## Introduction

One in three women in the United States (U.S.) will develop cancer in her lifetime, with breast and colorectal cancers being the most commonly diagnosed cancers among Hispanic women [1]. Hispanic women are more likely to be diagnosed with breast cancer at later stages, less likely to receive first course treatment meeting National Comprehensive Cancer Network standards (total mastectomy or breast-conserving surgery and radiation), more likely to be diagnosed with less-common molecular subtypes of breast cancer, and more likely to experience greater disparities in breast cancer mortality than their non-Hispanic White counterparts [2–5]. Additionally, Hispanic individuals are disproportionately affected by early-onset (diagnosis at < 50 years of age) colorectal cancer, are less likely to be diagnosed at earlier stages, and have a greater risk of not receiving guideline-concordant treatment, compared to the non-Hispanic White population [1, 6, 7]. The Hispanic population is rapidly growing in the U.S., with individuals of Mexican ethnic descent making up the largest proportion. Mexican descendants are a heterogenous group with respect to acculturation, and research has shown that greater acculturation and time spent in the U.S. are associated with increases in certain cancer risk factors, including the adoption of Western dietary patterns usually high in energy-dense and processed foods, and poor-quality diets [8–12].

Adherence to healthy dietary patterns may have a role in reducing risk of cancer, particularly colorectal and breast cancer in postmenopausal Hispanic women of Mexican descent [13]. Lifestyle factors, such as the adoption of healthy dietary patterns, may hold high potential to reduce risks of such cancers by way of reducing obesity. To date, 13 different cancers are associated with excess body weight, including breast and colorectal cancer [14]. Previous research has shown a greater risk of obesity associated with intake of a Western dietary pattern among Hispanic women [15], and among women of Mexican descent, two studies found increased odds of breast cancer associated with a Western diet [16] and a diet high in carbohydrates [17] With 50.9% of women of Mexican descent in the U.S. being classified as obese [18], identifying culturally-relevant dietary patterns that can reduce the risk of obesity and cancer is of great interest.

Prior studies have defined a traditional Mexican diet as one that centers on foods such as corn or maize, rice, legumes, and vegetables and that is low in added sugars, processed meats, processed foods and oils [19, 20]. Using participants from the Women’s Health Initiative (WHI) and an a priori derived traditional Mexican diet (MexD) score, prior analyses have shown that greater adherence to the traditional Mexican diet, as measured by the MexD score, was associated with lower systemic inflammation, insulin resistance, and obesity-related cancer mortality, although not associated with risk of metabolic syndrome [19, 21–23]. Several other studies have examined the association between different components of dietary patterns and risk of breast cancer among women of Mexican descent [17, 24–29]; however, to our knowledge, only two studies have investigated the relationship between a traditional Mexican dietary pattern and risk of cancer. One study among U.S. Hispanic women in the four corners region (Arizona, New Mexico, Colorado, and Utah) found that a traditional Mexican dietary pattern was associated with a 32% lower risk of breast cancer [30]. A previous study of Hispanic women in the WHI did not find an association between MexD scores and risk of any cancer, or obesity-related cancers [23]. To date, no study has evaluated the association of a traditional Mexican dietary pattern with risk of colorectal cancer in women of Mexican descent. The prospective WHI cohort provided the opportunity to examine the association of an a priori derived traditional Mexican diet score with risk of total cancer, and colorectal and breast specifically, among women who self-identified as being of Mexican ethnic descent.

## Methods

### Study population

The WHI enrolled 161,808 women aged 50 to 79 years at baseline (1993–1998) into the WHI Observational Study (OS), or one or more of three clinical trials, with enrollment occurring across the U.S. at 40 different clinical sites. Standardized questionnaires were administered at baseline to capture data on demographics, reproductive and medical histories, and lifestyle behaviors (including smoking status, alcohol use, and physical activity) while in-person interviews were used to collect current (baseline) menopausal hormone and medication use. Height and weight were also measured in the clinic at screening and were used to compute body mass index (BMI) as weight in kilograms (kg) divided by height in meters squared (m^2^). The WHI design and methods are available in greater detail elsewhere [31, 32]. From the WHI observational study and clinical trials, we excluded participants in a stepwise fashion who did not self-identify as being of Mexican descent (Mexican, Mexican American, or Chicano) (*n* = 159,115), with history of any cancer diagnosis other than non-melanoma skin cancer or missing cancer history at baseline (*n* = 206), missing energy intake at baseline (*n* = 2), and with an energy intake < 500 kcal or > 4,000 kcal or missing at baseline (*n* = 142) for a final sample size of 2,343. Participants were excluded for energy intake outside of the prespecified range as these values were considered improbable and potentially due to misreporting. Institutional review boards at each institution approved the WHI study, and all participants provided written informed consent.

### Traditional Mexican diet score

A self-administered food frequency questionnaire (FFQ) consisting of 122 separate items was administered at study baseline to WHI participants to obtain data on consumption of food and beverages in the prior three-month period. Validity of the WHI FFQ has not been shown to differ between non-Hispanic White and Hispanic women [33]. Design and development of the MexD score have been described in detail previously [19]. Briefly, the MexD score is calculated from 12 a priori defined food components or food groups (Table 1). A score of zero or one is assigned for each food component based on the cohort-specific median intakes. Beneficial foods, or foods more closely aligned with the hypothesized traditional Mexican diet, included corn tortillas, beans, soups, Mexican-mixed dishes, vegetables, fruits, rice, and whole-fat dairy. Women whose intake in each of these groups was below the cohort-specific median intake were assigned a value of zero, and women whose intake was at or above the cohort-specific median for each of these groups were assigned a value of one. Detrimental foods, or foods generally associated with a US diet, included oils, solid fats and added sugars, processed meats, and refined grains. Women whose consumption was below the median intake in each of these groups were assigned a value of one, and women whose consumption was at or above the median were assigned a value of zero. Scores for these food groups were then added together to calculate the total MexD score, with higher scores indicating greater adherence to the MexD pattern.

**Table 1:** Food components used to define traditional Mexican diet score on the Women’s Health Initiative Food Frequency Questionnaire.^a^

Beneficial foods	Detrimental foods
Corn tortillas (0.14) Beans (including refried) (0.11) Soups (0.12) Bean soups Vegetable soups Menudo/Tortilla soup Other soups Mexican-mixed dishes (0.20) Tamales (with or without meat) Chilaquiles Soft quesadillas Crispy quesadilla/Chile relleno Flautas/Taquitos Taco/Tostada Soft taco/Enchilada baked without oil Regular burrito/Enchilada Vegetables (3.04) Fruits (1.62) Rice (0.14) Whole-fat dairy (0.04)	Oils (0.49) Dressing Fries Chips Mayonnaise Solid fats and added sugars (1.60) Tang/Kool-Aid/Hi-C/Other fruit drinks Low-fat pizza Pizza Butter/Margarine/Oil on bread or tortillas Cottage and ricotta cheese Other cheese (Cheddar/Swiss/Cream cheese) Ice cream Pudding, custard, and flan Low-fat or non-fat frozen desserts Doughnut/Cake/Pastry/Pan Dulce Cookies Pumpkin and sweet potato pie Other pies/fried pastries/pastelitos (including fruit empanadas) Chocolate candy and candy bars Hard candy/Jam/Jelly/Honey/Syrup Regular soft drinks Low-fat milk Processed meats (0.32) Ground meat Lunch meat (Ham/Turkey/Lean meat) Other lunch meat (Bologna/Salami/Spam) Hot dogs/Chorizo/Sausage/Bratwurst Bacon/Breakfast sausage/Scrapple Refined grains (0.45) Macaroni and cheese/Lasagna/Noodles with cream sauce Spaghetti (with tomato or meat sauce) Biscuit/Muffin/Scone/Croissant White bread Pancakes and waffles

^a^Median intake (medium servings per day) for the sample population presented for each food group in parentheses

### Outcome ascertainment

Clinical outcomes were self-reported by participants, then adjudicated by trained physician adjudicators, as described elsewhere [34]. Briefly, participants were asked to report any physician-diagnosed new cancer, and additional documentation and copies of medical reports were requested following any self-reported incident cancer. Outcomes were first locally adjudicated using standardized criteria, then centrally reviewed. For cancer outcomes, pathology reports and hospital face sheets were reviewed by local adjudicators and additional documentation was sent for centralized review to determine cancer site, extent of disease, and tumor morphology. The WHI collected all invasive and in situ cancers of any histological subtype; however, for this study, only invasive cancers were analyzed as outcomes. Outcome data were available through February of 2020.

### Statistical analysis

MexD scores were grouped into tertiles (highest tertile representing greatest alignment with the traditional Mexican dietary pattern) and by median into a binary variable. We performed descriptive analyses to show the distribution of baseline demographic variables and cancer risk factors for women of Mexican ethnic descent by binary category of MexD scores. Multiple imputation by chained equations [35], using the “mice” package in R [36], was used to impute values for the following missing covariates, each of which had proportions of missing data under 30%: BMI, alcohol use, smoking history, recreational physical activity minutes per week, hormone therapy use, number of full-term pregnancies, mammography within two years before baseline, and NSAID use. We fit Cox proportional hazards models to calculate hazard ratios (HRs) and associated 95% confidence intervals (95% CIs) for the association between the MexD score at baseline and risk of all-cancer incidence, breast cancer, and colorectal cancer at follow-up. Models were fit on datasets with imputed covariates, with estimates pooled using Rubin’s rules [37]. Effect modification by BMI, preferred language, and age (50–59, 60–79) was examined using a Wald test. Potential confounders were identified a priori from the literature, and included age, total energy intake (kcal), BMI, history of diabetes, alcohol use, smoking history, physical activity, NSAID use, menopausal hormone therapy use, and preferred language (as a proxy measure of acculturation). Number of full-term pregnancies, Gail Model 5-year breast cancer risk score (Gail score) [38], and mammography were also potential confounders for breast cancer risk. History of colonoscopy was a potential confounder for colorectal cancer risk. In the majority of the imputed data sets, at least one category of preferred language had no cancer outcomes occurring for at least one of the tertiles of MexD score, therefore this covariate was excluded in the final model. Adjustment for BMI, physical activity, alcohol use, smoking status, history of diabetes, menopausal hormone therapy, Gail score, and recency of mammography did not change estimates by more than 10% and thus these variables were also excluded in final models to avoid over-adjustment with variables not associated with both exposure and outcome. Final models were adjusted for age, total energy intake, and participation in the WHI Dietary modification trial. Secondary analyses restricted to English speakers evaluated the association between the MexD score at baseline and risk of all-cancer incidence, breast cancer, and colorectal cancer at follow-up. All statistical tests were two-sided, and p-values less than 0.05 were considered statistically significant. All statistical analyses were conducted using R version 4.2.2 [39].

## Results

MexD scores ranged from zero to 12. Study participants with high MexD scores were more likely to prefer speaking Spanish, be never smokers, have higher recreational physical activity, and have four or more full-term pregnancies than participants with low MexD scores (Table 2). Median age at study baseline was 59 years and median BMI was 28 kg/m^2^ in women with both low, moderate, and high MexD scores. A total of 270 cancers, including 88 breast cancers and 37 colorectal cancers, were diagnosed over the course of a mean follow-up time of 14.4 years (range: 0.4–25.8 years) (Table 3).

**Table 2:** Distribution of demographic variables and cancer risk factors among women of Mexican ethnic descent (N = 2,343) at baseline in the Women’s Health Initiative (WHI) for low, moderate, and high traditional Mexican diet score categories

	Low MexD Scores 0-5 (N = 852)	Moderate MexD Scores 6-7 (N = 820)	High MexD Scores 8-12 (N = 671)	P-value
Age at baseline				
Mean (y) (SD)	59.4 (6.43)	60.0 (6.66)	60.1 (6.69)	0.07
Median [Min, Max]	59 [50, 79]	59 [50, 79]	59 [50, 79]	
Preferred language				
English	807 (94.7%)	680 (82.9%)	495 (73.8%)	< 0.001
Spanish	45 (5.3%)	140 (17.1%)	176 (26.2%)	
Body mass index at baseline				
Mean (kg/m.^2^) (SD)	29.3 (5.70)	29.8 (6.20)	28.8 (5.34)	0.006
Median [Min, Max]	28.3 [15.0, 54.3]	28.8 [17.3, 69.9]	28.0 [18.1, 66.3]	
Missing	7 (0.8%)	8 (1.0%)	4 (0.6%)	
Alcohol use				
Never	117 (13.7%)	152 (18.5%)	160 (23.8%)	< 0.001
Ever	726 (85.2%)	659 (80.4%)	505 (75.3%)	
Missing	9 (1.1%)	9 (1.1%)	6 (0.9%)	
Smoking status				
Never	514 (60.3%)	452 (55.1%)	466 (69.4%)	< 0.001
Ever	330 (38.8%)	281 (34.3%)	198 (29.5%)	
Missing	8 (0.9%)	7 (0.9%)	7 (1.0%)	
Minutes of recreational physical activity per week				
0-37.5	332 (39.0%)	255 (31.1%)	159 (23.7%)	< 0.001
> 37.5-173.3	256 (30.0%)	245 (29.9%)	205 (30.6%)	
> 173.3	237 (27.8%)	266 (32.4%)	233 (34.7%)	
Missing	27 (3.2%)	54 (6.6%)	74 (11.0%)	
Hormone replacement therapy				
Nonuser	366 (43.0%)	357 (43.5%)	300 (44.7%)	0.496
Past, < 5 years	66 (7.7%)	68 (8.3%)	62 (9.2%)	
Past, 5- < 10 years	21 (2.5%)	35 (4.3%)	21 (3.1%)	
Past, > 10 years	37 (4.3%)	28 (3.4%)	24 (3.6%)	
Current	360 (42.3%)	331 (40.4%)	264 (39.3%)	
Missing	2 (0.2%)	1 (0.1%)	0	
Number of term pregnancies				
None	78 (9.2%)	77 (9.4%)	58 (8.6%)	< 0.001
1	60 (7.0%)	58 (7.1%)	38 (5.7%)	
2	182 (21.4%)	143 (17.4%)	100 (14.9%)	
3	193 (22.7%)	159 (19.4%)	128 (19.1%)	
4	139 (16.3%)	149 (18.2%)	112 (16.7%)	
5 +	193 (22.7%)	228 (27.8%)	227 (33.8%)	
Missing	7 (0.8%)	6 (0.7%)	8 (1.2%)	
Recency of mammogram within 2 years before baseline				
Mammogram within 2 years	604 (70.9%)	575 (70.1%)	464 (69.2%)	0.866
No mammogram within 2 years	225 (26.4%)	221 (27.0%)	184 (27.4%)	
Missing	23 (2.7%)	24 (2.9%)	23 (3.4%)	
Ever had colonoscopy				
No	538 (63.1%)	460 (56.1%)	371 (55.3%)	0.109
Yes	282 (33.1%)	300 (36.6%)	222 (33.1%)	
Missing	32 (3.8%)	60 (7.3%)	78 (11.6%)	
NSAID use at baseline				
No	439 (51.5%)	426 (52.0%)	361 (53.8%)	0.685
Yes	167 (19.6%)	157 (19.1%)	122 (18.2%)	
Missing	246 (28.9%)	237 (28.9%)	188 (28.0%)	
Gail score^a^				
Mean (SD)	1.09 (0.687)	1.07 (0.601)	1.06 (0.653)	0.646
Median [Min, Max]	0.900 [0.470, 8.40]	0.900 [0.450, 6.70]	0.890 [0.450, 6.64]	
Diabetes at baseline				
No	777 (91.2%)	735 (89.6%)	600 (89.4%)	0.381
Yes	74 (8.7%)	85 (10.4%)	71 (10.6%)	
Missing	1 (0.1%)	0	0	

^a^Gail Model 5-year breast cancer risk score: predicts 5-year risk of breast cancer based on age, age at menarche, number of first-degree relatives with breast cancer, age at first live birth, number of breast biopsies, and presence of atypical hyperplasia in a previous biopsy

**Table 3:** Distribution of cancers diagnosed among women of Mexican ethnic descent in the Women’s Health Initiative (WHI) by cancer site.^a,b^

Cancer site	N (%)
Breast	87 (32.2)
Colorectal	29 (10.7)
Other gastrointestinal	6 (2.2)
Biliary/Gallbladder/Pancreas	9 (3.3)
Liver	5 (1.9)
Urinary (Bladder/Kidney/Ureter)	16 (5.9)
Endometrial	10 (3.7)
Ovary	10 (3.7)
Other Female Reproductive/Genital	6 (2.2)
Lung	16 (5.9)
Lymphoma (including Hodgkins)	12 (4.4)
Leukemia	8 (3.0)
Multiple Myeloma	9 (3.3)
Melanoma	6 (2.2)
Other	19 (7.0)
Unknown type	22 8.2)

^a^In participants with more than one cancer diagnosed during follow-up, only the first diagnosed cancer is included in the table

^b^Cancer sites with less than 5 participants are not displayed

Relative to the lowest tertile of MexD score, the highest tertile was associated with lower risk of all cancer incidence (HR: 0.67; 95% CI 0.49, 0.91) and colorectal cancer (HR: 0.38; 95% CI 0.14, 0.998), and a non-statistically significant lower risk of breast cancer (HR: 0.85; 95% CI 0.50, 1.45) (Table 4). When categorized as a binary variable, higher adherence to the traditional Mexican diet remained statistically significantly associated with a lower all-cancer incidence (HR: 0.79; 95% CI 0.61, 0.995), while association with lower risk of colorectal cancer (HR: 0.61; 95% CI 0.30, 1.24) did not. Each one unit increase in MexD score was associated with a 6% lower risk of all-cancer incidence (HR: 0.94; 95% CI 0.88, 0.99), a non-statistically significant 6% lower risk of breast cancer (HR: 0.94; 95% CI 0.85, 1.04), and a non-statistically significant 13% lower risk of colorectal cancer (HR: 0.87; 95% CI 0.74, 1.02).

**Table 4:** Risk of cancer at follow-up by traditional Mexican diet score among women of Mexican ethnic descent in the Women’s Health Initiative (WHI)

MexD score category (numeric score range)	All-cancer incidence		Breast cancer		Colorectal cancer	
	N (%)^a^	HR (95% CI)	N (%)^a^	HR (95% CI)	N (%)^a^	HR (95% CI)
Tertiles						
Low (0–5)	113 (41.9%)	1.0 (ref)	30 (34.1%)	1.0 (ref)	18 (48.6%)	1.0 (ref)
Moderate (6–7)	92 (34.1%)	0.82 (0.62, 1.08)	32 (36.4%)	1.01 (0.61, 1.67)	13 (35.1%)	0.71 (0.34, 1.50)
High (8–12)	65 (24.1%)	0.67 (0.49, 0.91)*	26 (29.5%)	0.85 (0.50, 1.45)	6 (16.2%)	0.38 (0.14, 0.998)*
P—trend		0.01		0.55		0.045
Binary						
Low (0–6)	161 (59.6%)	1.0 (ref)	47 (53.4%)	1.0 (ref)	24 (64.9%)	1.0 (ref)
High (7–12)	109 (40.4%)	0.79 (0.61, 0.995)*	41 (46.6%)	0.89 (0.58, 1.37)	13 (35.1%)	0.61 (0.30, 1.24)
Continuous	5.99 (2.13)	0.94 (0.88, 0.99)*	6.22 (2.24)	0.94 (0.85, 1.04)	5.68 (1.84)	0.87 (0.74, 1.02)

Adjusted for age, total energy intake at baseline (kcal), and participation in the WHI Dietary Modification clinical trial

^a^Mean (standard deviation) reported for continuous MexD score

*p-value less than 0.05

Due to a small number of events in women whose preferred language was Spanish, results are not presented as estimates would be too unstable; however, in secondary analyses restricted to English speakers, risk estimates were attenuated and were not statistically significant (Table 5). No evidence of effect modification by age, preferred language, or BMI was found, but results stratified by BMI are presented for all-cancer and breast cancer incidence in Supplementary Table 1.

**Table 5:** Risk of cancer at follow-up by traditional Mexican diet score among women of Mexican ethnic descent whose language preference was English in the Women’s Health Initiative (WHI)

MexD score category (numeric score range)	All-cancer incidence		Breast cancer		Colorectal cancer	
	N (%)^a^	HR (95% CI)	N (%)^a^	HR (95% CI)	N (%)^a^	HR (95% CI)
Tertiles						
Low (0–5)	106 (43.3%)	1.0 (ref)	30 (38.0%)	1.0 (ref)	16 (50.0%)	1.0 (ref)
Moderate (6–7)	84 (34.3%)	0.82 (0.62, 1.08)	27 (34.2%)	1.01 (0.61, 1.67)	11 (34.4%)	0.71 (0.34, 1.50)
High (8–12)	55 (24.4%)	0.67 (0.49, 0.91)*	22 (27.8%)	0.85 (0.50, 1.45)	5 (15.6%)	0.38 (0.14, 0.998)*
P—trend		0.01		0.55		0.045
Binary						
Low (0–6)	150 (61.2%)	1.0 (ref)	44 (55.7%)	1.0 (ref)	22 (68.8%)	1.0 (ref)
High (7–12)	95 (38.8%)	0.85 (0.65, 1.10)*	35 (44.3%)	0.95 (0.60 1.50)	10 (31.3%)	0.59 (0.27, 1.31)
Continuous	5.87 (2.08)	0.94 (0.88, 0.99)*	6.04 (2.22)	0.94 (0.85, 1.04)	5.68 (1.84)	0.87 (0.74, 1.02)

Adjusted for age, total energy intake at baseline (kcal), and participation in the WHI Dietary Modification clinical trial

^a^Mean (standard deviation) reported for continuous MexD score

*p-value less than 0.05

## Discussion

In this study of 2,343 postmenopausal women of Mexican descent, we found a significant association between greater adherence to a traditional Mexican diet, as measured by the MexD score, and lower all-cancer incidence and for colorectal cancer. We did not find evidence of an association between adherence to a traditional Mexican diet and risk of breast cancer.

To our knowledge, only two studies have previously reported the relationship between a traditional Mexican dietary pattern and risk of breast cancer among U.S. Hispanic women [23, 30]. The first, a case–control study of 757 breast cancer cases and 867 controls frequency-matched on ethnicity and age, found a Native Mexican dietary pattern (derived using factor analysis) to be associated with a 32% lower risk of breast cancer (OR: 0.68; 95% CI 0.55, 0.85) among all women and a non-statistically significant 39% lower risk (OR: 0.61; 95% CI 0.31, 1.20) among post-menopausal Hispanic women [30]. Among all premenopausal women, the observed association was greater in magnitude among women with a BMI < 25 kg/m^2^, while among all postmenopausal women, there was no difference in association by BMI [30]. The present study differs in its prospective design, our restriction to Hispanic women of Mexican descent, and the use of an a priori defined MexD pattern; key differences may have resulted in different findings.

The second, a previous study of Hispanic women in the WHI, did not find an association between MexD scores and risk of any cancer, or obesity-related cancers (meningioma and thyroid, esophageal, breast, stomach, pancreas, liver, gallbladder, kidney, multiple myeloma, uterine, ovarian, and colorectal cancers) [23]. These discrepancies may have resulted from one main difference in analysis: the previous WHI study included all Hispanic women, regardless of specific ethnic origin, while ours was restricted to women of Mexican descent, the group for whom a traditional Mexican diet is likely more common and culturally relevant.

Among women of Mexican descent specifically, we are only aware of two studies that examined other characteristics of diet in relation to breast cancer risk. One case–control study of women from northern Mexico found greater odds of breast cancer associated with a Western dietary pattern and lower odds of breast cancer associated with a prudent dietary pattern characterized by increased consumption of vegetables, legumes, and corn [16]. These associations were found for both pre- and postmenopausal women. In another case–control study of premenopausal Mexican women, high carbohydrate consumption was associated with greater odds of breast cancer [17].

Previous research in the WHI cohort found a reduced risk of systemic inflammation and insulin resistance associated with higher MexD scores among women of Mexican descent [19]. Adherence to the MexD may reduce risk of colorectal cancer by reducing levels of inflammation in the body. Consistent with this hypothesis, one meta-analysis identified higher risks of colorectal cancer associated with greater inflammatory potential of diet, as measured by the Dietary Inflammatory Index [40]. As we did not observe a relationship between MexD score and breast cancer risk, it is possible that the anti-inflammatory effects of the MexD are more pronounced in the colon, or are more important for the prevention of select cancer sites, including colorectal cancer. Furthermore, the MexD is characterized by a lower consumption of processed meats, and there is strong evidence that processed meat increases the risk of colorectal cancer [41].

The main strength of this study is its prospective design, unlike most prior studies that retrospectively examined the link between MexD and cancer. Additionally, this study took advantage of the well-established WHI cohort, which captured a wide range of breast cancer risk factors for postmenopausal women across the U.S. using validated methods [32]. This study also has limitations. The sample size for Mexican women was small relative to the overall WHI. Beyond secondary analyses restricted to English speakers, we were not able to explore the influence of acculturation (using language preference as a proxy) with sufficient power as only nine participants (9.9%) with breast cancer at follow-up preferred using Spanish to communicate. Among Hispanic women in the U.S., acculturation has been associated with poorer diet quality [8–11] and with increased risk of breast and other cancers [42, 43], and these relationships may have impacted our findings in this highly acculturated sample. We also were unable to adjust for recency of smoking status, due to the small sample size of current smokers at baseline; however, estimates from a sensitivity analysis excluding current smokers were not meaningfully different. Additionally, dietary exposures were measured at baseline, and we did not have data on longitudinal dietary exposures, which prevented us from assessing change of dietary habits; however, given the extended time it takes for most cancers to develop, measuring diet at baseline increases the chance that the measured diet predates initial development of the cancer. Finally, we did not have sufficient sample size to examine this relationship by molecular subtype of breast cancer, so findings may only be generalizable to women with the more common estrogen receptor positive, human epidermal growth factor receptor 2 (HER2) negative, breast cancer.

Overall, we found that greater adherence to the MexD was associated with a lower risk of all-cancer incidence and colorectal cancer but not breast cancer among a cohort of postmenopausal U.S. women of self-identified Mexican ethnic origin who participated in the WHI. Future randomized trials are needed to confirm whether a traditional Mexican diet can prevent colorectal cancer, given the rise in early-onset cases and the growing Hispanic population in the U.S.

### Supplementary Information

Below is the link to the electronic supplementary material.

Supplementary file1 (DOCX 17 KB)

Supplementary file2 (DOCX 15 KB)

## Data Availability

The data underlying this article are provided by the Women’s Health Initiative upon approval of reasonable proposal.
