# Supplementary material for: Traditional Mexican dietary pattern and cancer risk among women of Mexican descent
Source: Cancer Causes Control. 2024 Feb 2;35(6):887–96. doi: 10.1007/s10552-024-01849-5 (PMC11129927; doi:10.1007/s10552-024-01849-5)
Supplement: Supplementary file 1 — Supplementary file1 (DOCX 17 KB) [file 10552_2024_1849_MOESM1_ESM.docx]

Supplementary Table 1. Risk of cancer at follow-up by traditional Mexican diet score among women of Mexican ethnic descent in the Women’s Health Initiative (WHI), stratified by body mass index (BMI).^1^

| **MexD score category (numeric score range)** | **All-cancer incidence** | | **Breast cancer** | |
| --- | --- | --- | --- | --- |
|  | **N (%)^2^** | **HR (95% CI)** | **N (%)^2^** | **HR (95% CI)** |
| **BMI 18.5-24.9** |  |  |  |  |
| Tertiles |  |  |  |  |
| Low (0-5) | 24 (43.6%) | 1.0 (ref) | 6 (40.0%) | 1.0 (ref) |
| Moderate (6-7) | 15 (27.3%) | 0.67 (0.34, 1.29) | 5 (33.3%) | 0.72 (0.18, 2.92) |
| High (8-12) | 16 (29.1%) | 0.84 (0.43, 1.64) | † | † |
| Binary |  |  |  |  |
| Low (0-6) | 32 (58.2%) | 1.0 (ref) | 8 (53.3%) | 1.0 (ref) |
| High (7-12) | 23 (41.8%) | 0.86 (0.49, 1.51) | 7 (46.7%) | 0.72 (0.22, 2.38) |
| Continuous | 5.89 (2.27) | 0.92 (0.80, 1.05) | 5.80 (2.14) | 0.80 (0.59, 1.09) |
| **BMI 25-29.9** |  |  |  |  |
| Tertiles |  |  |  |  |
| Low (0-5) | 40 (38.8%) | 1.0 (ref) | 10 (27.8%) | 1.0 (ref) |
| Moderate (6-7) | 35 (34.0%) | 0.91 (0.58, 1.45) | 15 (41.7%) | 1.40 (0.61, 3.24) |
| High (8-12) | 28 (27.2%) | 0.70 (0.42, 1.14) | 11 (30.6%) | 0.80 (0.32, 1.99) |
| Binary |  |  |  |  |
| Low (0-6) | 56 (54.4%) | 1.0 (ref) | 16 (44.4%) | 1.0 (ref) |
| High (7-12) | 47 (45.6%) | 0.83 (0.56, 1.24) | 20 (55.6%) | 1.14 (0.57, 2.29) |
| Continuous | 6.16 (2.16) | 0.94 (0.86, 1.03) | 6.28 (2.31) | 0.91 (0.78, 1.07) |
| **BMI ≥ 30** |  |  |  |  |
| Tertiles |  |  |  |  |
| Low (0-5) | 48 (44.4%) | 1.0 (ref) | 13 (36.1%) | 1.0 (ref) |
| Moderate (6-7) | 39 (36.1%) | 0.75 (0.49, 1.15) | 12 (33.3%) | 0.85 (0.38, 1.95) |
| High (8-12) | 21 (19.4%) | 0.62 (0.37, 1.04) | 11 (30.6%) | 1.16 (0.49, 2.74) |
| Binary |  |  |  |  |
| Low (0-6) | 69 (63.9%) | 1.0 (ref) | 22 (61.1%) | 1.0 (ref) |
| High (7-12) | 39 (36.1%) | 0.74 (0.50, 1.10) | 14 (38.9%) | 0.81 (0.40, 1.64) |
| Continuous | 5.93 (2.03) | 0.95 (0.86, 1.04) | 6.44 (2.14) | 1.06 (0.90, 1.26) |

Adjusted for age, total energy intake at baseline (kcal), and participation in the WHI Dietary Modification clinical trial.

1. Low number of colorectal cancer cases (<5) in the majority of categories prevented the display of results for colorectal cancer incidence.

2. Mean (standard deviation) reported for continuous MexD score

* p-value less than 0.05

† Less than 5 cancer cases, data not shown
