# Supplementary material for: Traditional Mexican dietary pattern and cancer risk among women of Mexican descent
Source: Cancer Causes Control. 2024 Feb 2;35(6):887–96. doi: 10.1007/s10552-024-01849-5 (PMC11129927; doi:10.1007/s10552-024-01849-5)
Supplement: Supplementary file 2 — Supplementary file2 (DOCX 15 KB) [file 10552_2024_1849_MOESM2_ESM.docx]

This study acknowledges the following WHI investigators:

**Program Office**: (National Heart, Lung, and Blood Institute, Bethesda, Maryland) Jacques Rossouw, Shari Ludlam, Joan McGowan, Leslie Ford, and Nancy Geller

**Clinical Coordinating Center**: (Fred Hutchinson Cancer Research Center, Seattle, WA) Garnet Anderson, Ross Prentice, Andrea LaCroix, and Charles Kooperberg

**Investigators and Academic Centers**: (Brigham and Women's Hospital, Harvard Medical School, Boston, MA) JoAnn E. Manson; (MedStar Health Research Institute/Howard University, Washington, DC) Barbara V. Howard; (Stanford Prevention Research Center, Stanford, CA) Marcia L. Stefanick; (The Ohio State University, Columbus, OH) Rebecca Jackson; (University of Arizona, Tucson/Phoenix, AZ) Cynthia A. Thomson; (University at Buffalo, Buffalo, NY) Jean Wactawski-Wende; (University of Florida, Gainesville/Jacksonville, FL) Marian Limacher; (University of Iowa, Iowa City/Davenport, IA) Jennifer Robinson; (University of Pittsburgh, Pittsburgh, PA) Lewis Kuller; (Wake Forest University School of Medicine, Winston-Salem, NC) Sally Shumaker; (University of Nevada, Reno, NV) Robert Brunner

**Women’s Health Initiative Memory Study:** (Wake Forest University School of Medicine, Winston-Salem, NC) Mark Espeland

*For a list of all the investigators who have contributed to WHI science, please visit:* [*https://s3-*](https://s3-us-west-2.amazonaws.com/www-whi-org/wp-content/uploads/WHI-Investigator-Long-List.pdf) [*us-west-2.amazonaws.com/www-whi-org/wp-content/uploads/WHI-Investigator-Long-List.pdf*](https://s3-us-west-2.amazonaws.com/www-whi-org/wp-content/uploads/WHI-Investigator-Long-List.pdf)
